# Evoked Hemodynamic Response Estimation to Auditory Stimulus Using Recursive Least Squares Adaptive Filtering with Multidistance Measurement of Near-Infrared Spectroscopy

**DOI:** 10.1155/2018/7609713

**Published:** 2018-03-29

**Authors:** Yan Zhang, Xin Liu, Dan Liu, Chunling Yang, Qisong Wang, Jinwei Sun, Kuanquan Wang

**Affiliations:** ^1^School of Electrical Engineering and Automaton, Harbin Institute of Technology, Harbin 150001, China; ^2^School of Computer Science and Technology, Harbin Institute of Technology, Harbin 150001, China; ^3^School of Transportation Science and Engineering, Harbin Institute of Technology, Harbin 150090, China

## Abstract

The performance of functional near-infrared spectroscopy (fNIRS) is sometimes degraded by the interference caused by the physical or the systemic physiological activities. Several interferences presented during fNIRS recordings are mainly induced by cardiac pulse, breathing, and spontaneous physiological low-frequency oscillations. In previous work, we introduced a multidistance measurement to reduce physiological interference based on recursive least squares (RLS) adaptive filtering. Monte Carlo simulations have been implemented to evaluate the performance of RLS adaptive filtering. However, its suitability and performance on human data still remain to be evaluated. Here, we address the issue of how to detect evoked hemodynamic response to auditory stimulus using RLS adaptive filtering method. A multidistance probe based on continuous wave fNIRS is devised to achieve the fNIRS measurement and further study the brain functional activation. This study verifies our previous findings that RLS adaptive filtering is an effective method to suppress global interference and also provides a practical way for real-time detecting brain activity based on multidistance measurement.

## 1. Introduction

Functional near-infrared spectroscopy (fNIRS) has been demonstrated to have the potential to discover hemodynamic variations within the cortex [1, 2]. It is an increasingly popular technology for brain activity assessment due to its several advantages over other techniques (fMRI, EEG, etc.) for portability, low cost of the measurement equipment, and low restraints of subjects [3, 4]. In real applications, the useful signal is in the deeper regions of the brain and the strong mixture between the physiological interference and the brain activity response presents significant challenges in signal extraction [5]. The suppression of this kind of physiological interference is very important to accurately access the brain function in fNIRS measurement.

The physiological interference is mainly from the perturbation caused by heartbeat, respiratory, and blood pressure variation. All of these interference sources are located both in the vasculature of the superficial layer of the brain and inside the brain and are often low correlated with the functional response of the brain activity [6]. Previous attempts have been made to reduce such kind of interference and retrieve the brain function response. Various approaches have been investigated to extract hemodynamic response from fNIRS recordings in the past either based on single distance or multidistance fNIRS instruments. Low-pass filtering (LPF) or band-pass filtering (BPF) technique is a simple method to remove the interference caused by cardiac oscillations. LPF is particularly effective and easy to implement for the single distance measurement. However, LPF is not effective at removing the specific physiological noise signal such as respiratory and blood pressure fluctuations since there are partial overlap in the frequency of these fluctuations and the low frequency components of the hemodynamic response. Based on the single channel probe arrangement, empirical mode decomposition (EMD) algorithm together with Hilbert transform was presented to be an effective method for suppressing the physiological interferences from the fNIRS signal. This methodology has the advantage of simplicity in instrument design and the possibility for the application in optical topography [7].

Recently, multidistance measurement is extensively adopted to eliminate physiological interference in fNIRS data based on the use of additional short source-detector separation [8, 9]. Owing to the direct relation between source-detector separation and depth reached by photons within tissues, the short source-detector separation measurement is mostly sensitive to the superficial layer hemodynamic changes, and the long source-detector separation measurement is used to observe the hemodynamic changes both in the superficial layer and in the cerebral cortex. The short separation measurement is used as the reference signal, and the long separation measurement is treated as the target signal [10]. Next, signal from this reference is employed to estimate global interference, which is then removed from the target signal to obtain the stimulus-evoked brain activity [11]. Many different algorithms have been implemented to improve estimation of hemodynamic response from fNIRS measurements based on the use of the multidistance measurement. Least mean squares-based adaptive filtering has previously presented the potential to suppress physiological interference either for simulated signal or for real human data [9, 12]. Other novel effective methods [3, 13, 14] relying on combining different algorithms were recently used for reducing the physiological interference and improving the signal to noise ratio of the evoked brain activity signal.

Previously, we have developed recursive least squares (RLS) adaptive filtering to remove physiological interferences based on a multidistance probe configuration [4]. The RLS-based algorithm appeared to have significant power to suppress the physiological interference in the synthetic data, yielding small MSE and fast convergence. Monte Carlo simulations suggest that signal from short detector actually dominates the major component of the physiological interference, thus making it an appropriate reference measurement for adaptive filtering-based interference cancellation. Our Monte Carlo simulation results suggest that this method is very effective in reducing mean squared error (MSE) between the estimated brain activity and the real simulated response. This algorithm is fast enough to recover the brain activity for real-time applications. However, its suitability and performance on human data remains to be evaluated. Here, we describe the experiment of evoked auditory response detection as a further study of our methodology. A multidistance probe with continuous wave fNIRS technology is devised to implement the NIRS measurement. To evaluate the performance of the RLS algorithm in the *in vivo* experiment, the power spectral density (PSD) and contrast to noise (CNR) are analyzed in our study.

## 2. Materials and Method

### 2.1. NIRS Instrumentation

We used two LEDs (SMT760/850), at 760 and 850 nm, respectively, and three monolithic photodiodes as detectors (OPT101, with on-chip transimpedance amplifier; Burr-Brown Inc., Tucson, Arizona). The two LEDs and three detectors are typically configured as a multidistance probe. One of the two LEDs is selected to emit the light, and two of the three detectors are chosen to obtain the output light according to different subject, with the near detector sensing systemic physiological fluctuations and the far detector additionally measuring deeper tissue hemodynamic response.

**Figure 1:**
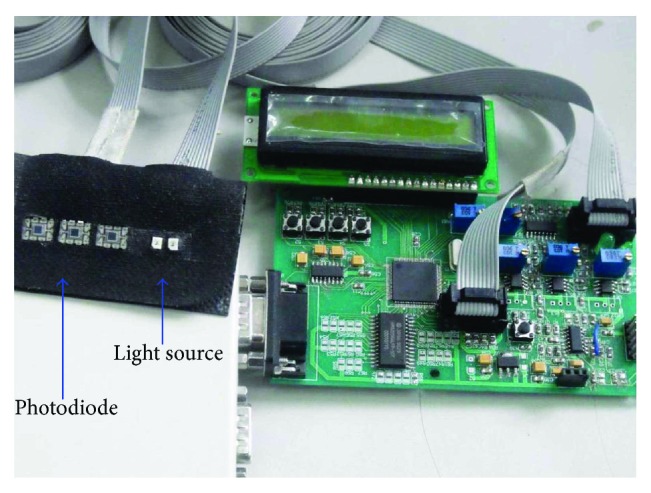
Experimental apparatus for brain activity measurement with NIRS.

The optical channels work in frequency division multiplexing mode, so that the signal from the two light sources can be separated and the ambient light can be removed. Once the light pass through the head and are detected by the detectors, they are separated by demodulating and then amplified. To reduce the noise, a 10 Hz low-pass filter is designed at each detector channel before the signal is digitized and sampled into the memory. A calibration procedure is required to correct any signal offset such as dark current.  Figure 1 shows the experimental apparatus for brain activity measurement with near-infrared spectroscopy.

### 2.2. Experiment

The auditory stimulation task was performed on a healthy, 21-year-old female, with no known history of neurological, psychiatric, auditory, or visual problems. In the experiment, the subject had been given a verbal agreement to participate in the test study. Optical data was collected using the NIRS system based on the auditory block design experiment. The block design experiments are always adopted by the fNIRS groups to analyze the hemodynamic response. The experiment is implemented based on some published literatures [7, 9]. The concentration changes of the oxyhemoglobin, Δ[HbO_2_], and deoxyhemoglobin, Δ[HHb], can be observed in the auditory cortex of the subject. In the experiment, the sound stimulation was prepared by using GoldWave audio editor software. First, we select concise and powerful music as the sensitive stimulus according to the opinion of the participant. The editing procedure is done by cutting down 20 seconds in the climax part of the music and followed by the silent mode for 20 seconds. The whole block is composed of the “stimulus” stage and the “resting” stage. The “resting” stage and the “stimulus” stage are repeated alternately during the experiment. One about 7-minute run of 10 blocks was presented, and the auditory stimulation frequency was 0.025 Hz.

**Figure 2:**
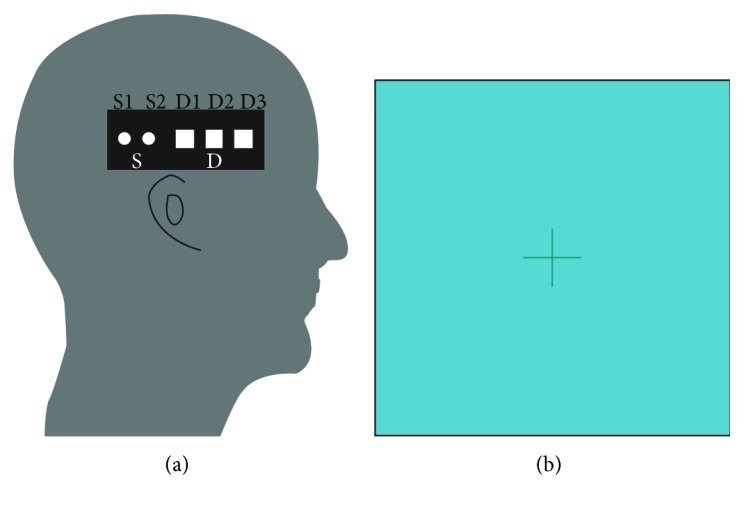
Diagram of auditory block-design experiment. (a) Probe position on the head. (b) Central fixation cross for gaze.

The probe location depends on the experiment type and the corresponding response area. For the auditory stimulus, the response area should be temporal region. Left temporal region is at approximately position T3 and the right temporal region is at approximately position T4 according to the International 10–20 system [15]. In our experiment, the sensor probe is placed on the upper side of the head, located near the T4 position of the international standard 10–20 guiding system (Figure 2). The subject listens carefully to the music using the earphones, and the eyes focused on the central marking area of the screen. Then the participant can pay attention to the auditory stimulus. The combined light of two wavelengths was in contact with tissue in one source location (S2 in Figure 2(a)), and exiting light was collected from two detector locations, 13 mm and 35 mm from the source, respectively (D1 and D3 in Figure 2(a)).

In the experiment, in order to reduce the artifacts caused by head movement, the subjects lean on the chair and the sensor probe was fixed to the test area to minimize head swaying. In order to ensure that the participant is not disturbed by the outside sound, the experiment is carried out in the quiet and dark laboratory. Soft black Velcro was also used to absorb stray light beneath the probe. Before the stimulus test, the baseline stage is carried out at least 200 seconds until the subject is calm. There is no stimulation during this period and the subject is required to keep quiet and calm.

### 2.3. Data Analysis

In the experiment, we consider the unstable light intensity of LED. Therefore, we first correct the optical data according to the light intensity drift. Then the light density changes of each channel are calculated on the basis of the measured data at the baseline stage. The light density changes obtained by the modified Lambert-Beer law are then converted to the concentration changes of HbO_2_ and HHb. The time series of concentration changes of oxyhemoglobin were analyzed by adaptive filtering method. The HbO_2_ concentration obtained by S2-D1 and S2-D3 was used as the reference signal and the target signal of the adaptive filtering input, respectively. The time sequence of HHb concentration change was processed by the same method. RLS adaptive filtering takes advantage of the finite impulse response (FIR) described earlier in our previous paper, and the filter order is *N* = 16. The recursive least squares method is used as the optimization algorithm for the coefficient of the filter, and it is calculated point by point. The forgetting factor *X* is 0.999, and the initial filter coefficient is set to [0 0 0]^T^. The method is based on a linear mapping relationship between S2-D1 and S2-D3, and the mapping relation depends on the sampling values near the time point and adjusts with time adaptively.

Another problem is that the magnitudes of Δ[HbO_2_] and Δ[HHb] are underestimated due to the so-called partial volume effect (PVE) [16]. Monte Carlo simulations have been previously used to estimate the ratio of the optical path length in the activated volume to the optical path length in the sampling volume. After adaptive filtering, partial volume effect factor (PVEF) is introduced to compensate partial volume effect in order to obtain relatively accurate results of cerebral cortex hemodynamic changes. Because the value of PVEF cannot be obtained accurately in the actual measurement, the PVEF is set to 9.15 by our Monte Carlo simulation. For the results of adaptive filtering, the spectrum components are analyzed by power spectral density (PSD), including hemodynamic response and physiological interference. The metric of contrast to noise is calculated before and after RLS adaptive filtering according to the PSD data.

## 3. Results

### 3.1. HbO_2_ Changes during Auditory Stimulation

**Figure 3:**
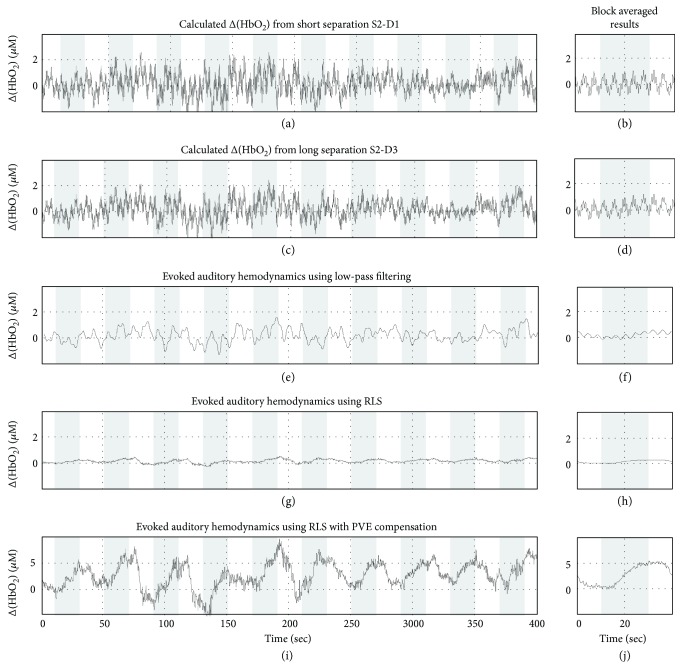
RLS adaptive filtering to remove physiological interference. (a) Reference measurements of Δ[HbO_2_] calculated from S2-D1 with 13 mm source-detector separation and (b) its block averaged result. (c, d) Target measurements from S2-D3 with 35 mm source-detector separation. (e, f) Low-pass filtering result for the target measurements. (g, h) RLS adaptive filtering results for target measurements. (i, j) Adaptive filtering result with PVE compensation.

The concentration changes of oxyhemoglobin, Δ[HbO_2_], and the concentration changes of deoxyhemoglobin, Δ[HHb], were filtered using the RLS algorithm, and the results are presented in Figure 3. We show both the time series of the calculated concentrations (the first column) and their block averaged results (the second column). The block-averaged results were achieved by averaging the stimulation and the rest periods within 10-epoch blocks. Hereinto, Figure 3(a) shows the Δ[HbO_2_] calculated with the reference channel S2-D1, and Figure 3(b) shows the block averaged result. Figure 3(c) shows the Δ[HbO_2_] calculated with the target channel S2-D3, and Figure 3(d) shows the block averaged result. Neither the raw time series nor the block average result shows any obvious expected signal change in Figures 3(a) and 3(b). It is concluded that the detected light is not sensitive to the deep region of the head. From Figures 3(c) and 3(d), we hope to observe the tendency of oxyhemoglobin concentration corresponding to the stimulus, namely, the concentration of oxyhemoglobin increases at the beginning of the auditory stimulation and gradually returned to the normal value after the stimulation. However, the tendency of Δ[HbO_2_] is not obvious during the whole block experiment either from the calculated value or the block average result. The physiological interference is very large, and the brain activity signal is not easy to be retrieved. Thus, we calculate the correlation coefficient between the reference signal from S2-D1 and the measurement signal from S2-D3 during the baseline period. The correlation coefficient is 0.89, which means that the data in the reference channel and the physiological interference in the measurement channel have a high correlation. Therefore, we can adopt adaptive filtering to suppress the physiological interference.

To compare with the adaptive filtering method, the raw signal is low-pass filtered at a cut-off frequency of 0.125 Hz. The Δ[HbO_2_] with low-pass filtering and its block averaged results are shown in Figures 3(e) and 3(f). The low-pass filtering suppresses a large proportion of high-frequency physiological interference. However, the periodicity of brain activity is not obvious. The Δ[HbO_2_] after RLS adaptive filtering is shown in Figures 3(g)–3(j). Figures 3(g) and 3(h) are Δ[HbO_2_] obtained by RLS adaptive filtering and the block averaged results. Figures 3(i) and 3(j) are Δ[HbO_2_] after compensating with the partial volume effect and its block averaged results. That is, the results shown in Figures 3(g) and 3(h) were corrected with PVEF using the results in Figures 3(c) and 3(d). The physiological interferences in Figures 3(g) and 3(h) were significantly suppressed. It can be seen from the entire time series of Δ[HbO_2_] and their block averaged results that the oxyhemoglobin concentration increases significantly at the initial presentation of the musical stimulus and gradually returned to normal value after the stimulus. For the data corrected with PVEF, the tendency is more obvious. From the block averaged results, it can be seen that at the initial time of stimulation, the hemodynamic response is delayed by about 4 seconds relative to the auditory stimulus, and the hemodynamic changes do not disappear immediately after the stimulus finished, with a delay of about 6 seconds and then gradually return to the normal state. By comparing the results of low-pass filtering and RLS adaptive filtering, the expected signal change can be seen from Figures 3(g)–3(j). It is obvious that low-pass filtering can only eliminate high-frequency interference but RLS algorithm can effectively suppress physiological noise.

**Figure 4:**
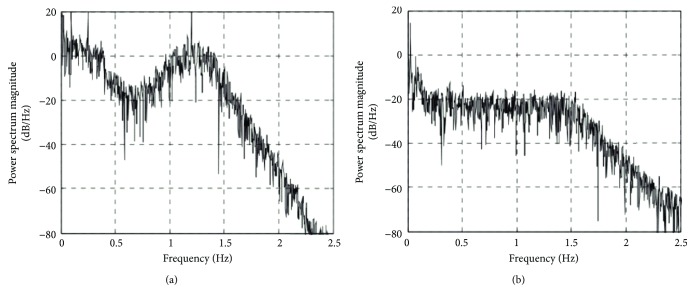
Power spectrum intensity of HbO_2_ (a) before RLS adaptive filtering and (b) after RLS adaptive filtering.

Unlike in our previous Monte Carlo simulation study, a truly rigorous evaluation of RLS method *in vivo* requires an uncontaminated evoked brain activity response signal, which, unfortunately, is unavailable. Therefore, in order to further investigate the validity of RLS adaptive filtering algorithm in hemodynamic parameter measurement, the power spectral density before and after filtering is analyzed.  Figure 4(a) shows the power spectral distribution of the original signal. It can be seen that there is a significant peak around 1.2 Hz in the frequency range of 0–2.5 Hz, which corresponds to the body's cardiac cycle.  Figure 4(b) is the result of RLS adaptive filtering, and the high frequency physiological interference is significantly suppressed compared with the amplitude of the power spectrum before filtering.

To further evaluate the effectiveness of the RLS algorithm, the contrast noise ratio (CNR) is calculated here to analyze the processing results. Here, we use the power spectral density to calculate the CNR before and after filtering. In the *in vivo* experiments, the brain activity signal and noises cannot be separated completely, and then the energy of signal and noise is obtained by integrating the power spectral density of “signal frequency band” and “noise frequency band” by using the method in the literature [13]. The CNR is then calculated as the square root of “signal energy” and “noise energy.” For the auditory block-design experiment, each block time is 40 seconds. The brain function signal is not a simple sinusoidal signal, and there are harmonic components. Here, the base band frequency is 0.025 Hz and the second harmonic frequency is 0.05 Hz. Thus, we determine the frequency domain near the base band 0.019–0.031 Hz, and the second harmonic frequency 0.044–0.056 Hz as a signal frequency band, and other frequency values in the range of 0–5 Hz as the noise band. Although this method inevitably introduces errors, it is a relatively valid assessment method for the *in vivo* experiments. In particular, the power spectral density is not calculated from a single block but from 4000 points over the entire time series. Through the comparison, the CNR for original signal is 30.31%, and the CNR of the filtered data is improved to 146.38%. The result demonstrates that RLS adaptive filtering method based on multidistance measurement can effectively reduce physiological interference.

### 3.2. HHb Changes during Auditory Stimulation

**Figure 5:**
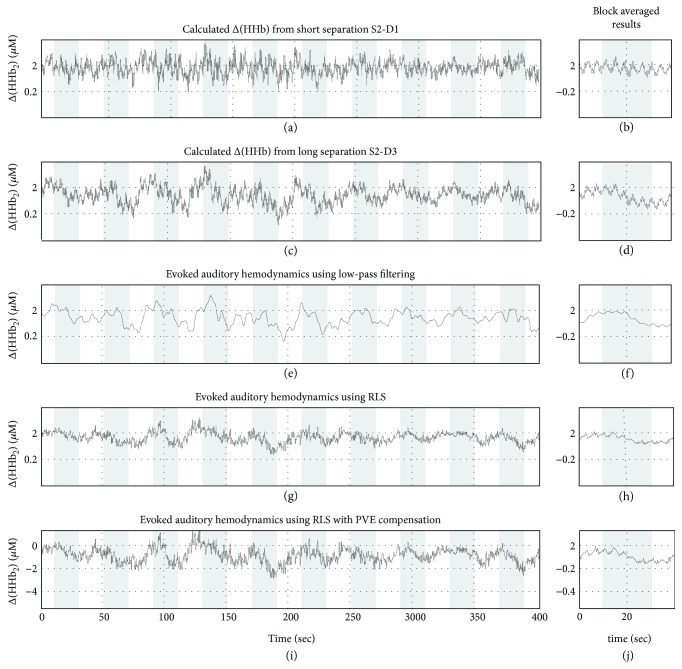
RLS adaptive filtering to remove physiological interference. (a) Reference measurements of Δ[HHb] calculated from S2-D1 with 13 mm source-detector separation and (b) its block averaged result. (c, d) Target measurements from S2-D3 with 35 mm source-detector separation. (e, f) Low-pass filtering result for the target measurements. (g, h) RLS adaptive filtering results for target measurements. (i, j) Adaptive filtering result with PVE compensation.

Figure 5 is the change in the concentration of HHb measured in the auditory block experiment. Figures 5(a)–5(j) present the equivalent result of Figures 3(a)–3(j), respectively, but for HHb. From Figures 5(a)–5(d), we can see that the measured HHb concentration variation is smaller than that of HbO_2_, and the result is in accordance with the literature [17]. The measured results in Figures 5(c) and 5(d) can better reflect the hemodynamic response, but the trend is opposite to HbO_2_. Such results are different from Figures 3(c) and 3(d), which indicate that physiological interference has relatively low influence on deoxyhemoglobin concentration change measurement. It can be explained that physiological interference is mostly from arterial blood, and arterial blood has a higher proportion of HbO_2_ than venous blood. Therefore, the physiological interference introduced by HbO_2_ is more significant. Comparing with the results of low-pass filter (Figures 5(e) and 5(f)), RLS filtering results (Figures 5(g) and 5(h) and the PVEF correction value of RLS filter results (Figures 5(i) and 5(j)) do not improve the quality of measurement signal significantly.

**Figure 6:**
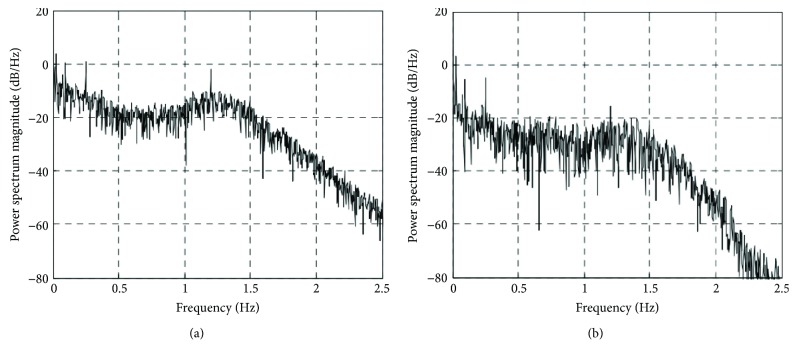
Power spectral density of HHb. (a) Before RLS adaptive filtering. (b) After RLS adaptive filtering.

Similarly, in order to analyze the effectiveness of RLS filtering for HHb detection from the frequency domain, the power spectral density of HHb is presented in Figure 6. Figures 6(a) and 6(b), respectively, is the power spectrum before and after the filtering in the 0–2.5 Hz range. By analyzing the contrast noise ratio of HHb, it is found that the contrast noise ratio before HHb filtering is 86.43%. Compared with the contrast noise ratio of HbO_2_, we can see that the physiological interference of HHb is relatively low, which is also seen from Figure 6(a). That is, the peak value of power spectrum density at 1.2 Hz is not obvious. After filtering, the contrast noise ratio is 88.84%, which indicates that the improvement of HHb using RLS adaptive filtering is not obvious.

## 4. Discussion

The fNIRS can be used to noninvasively monitor cerebral functional hemodynamics. However, in real situations, fNIRS recordings are often corrupted by interference engendering from ongoing physiological activities which occurs in both superficial and brain tissue layers. The suppression of this interference is important for reliable extraction of brain activity measurements because these physiological fluctuations can significantly degrade the signal quality. Therefore, the estimation of evoked hemodynamic response from fNIRS signal still remains a challenging task.

Adaptive filtering has been widely used to help identify and separate the interference components in fNIRS data. Some of them use auxiliary physiological measurements such as the pulse oximeter, electrocardiogram (ECG), chest band respirometer, spirometer, and capnograph [18]. This method is effective in reducing global interference, but the indispensability of additional equipments is the limitation of its application. The RLS algorithm thus presents the potential to obtain the evoke hemodynamic response in fNIRS data based on multidistance measurement.

In our previous study, RLS has also been explored for physiological interference reduction with the synthetic fNIRS data. The RLS algorithm iteratively computes the updated estimate of the filter coefficients upon the arrival of new data. The MSE, the convergence rate, the optimum source detector arrangement, and sensitivity of superficial layer thicknesses were delicately discussed. The use of NIRS multidistance probe arrangements for adaptive filtering depends on the assumptions that signal acquired from the near detector are not caused by brain activity and signal acquired from far detector are sufficiently sensitive to brain activity. In the *in vivo* experiment, signal from near detector can present the physiological interference since the correlation coefficient between the near-detector signal and the far-detector signal measured in the baseline stage is high.

We are currently working on the evaluation of the RLS adaptive filtering on the real human data. It is found that the physiological interference in HHb is less obvoius than that in HbO_2_. Such phenomenon can be explained that the blood in the venous compartments exhibited little physiological interference, whereas the physiological interference in HbO_2_ present in all compartments was substantial. It also can be seen in Figures 4 and 6 that the energy of the noise frequency band in power spectral density is smaller for HHb than that for HbO_2_. The CNR in evoked hemodynamic response improved substantially for HbO_2_ and that for HHb. We consider that this occurred due to excessive physiological interference of the overlying layers and predominantly in HbO_2_, a challenge which was effectively resolved by RLS adaptive filtering.

## 5. Conclusions

The RLS adaptive filtering is a promising approach to analyze evoked hemodynamic response. The evaluation of this method is implemented based on the multidistance measurement. An auditory block experiment was designed to analyze the changes in hemodynamic parameters in the temporal lobe region by exerting musical stimulus. The modified Lambert-Beer law is used to convert the optical data to the hemodynamic parameters in the reference channel and in the target channel. It is found that the hemodynamic parameters in the measurement channel have obvious physiological interference and the evoked hemodynamic response is always masked by the interference. RLS adaptive filtering is used to extract hemodynamic parameters. By analyzing the PSD and the CNR, we find that RLS adaptive filter can suppress physiological interference, especially for the presence of obvious interference of oxyhemoglobin.
